# Six-SOMAmer Index Relating to Immune, Protease and Angiogenic Functions Predicts Progression in IPF

**DOI:** 10.1371/journal.pone.0159878

**Published:** 2016-08-04

**Authors:** Shanna L. Ashley, Meng Xia, Susan Murray, David N. O’Dwyer, Ethan Grant, Eric S. White, Kevin R. Flaherty, Fernando J. Martinez, Bethany B. Moore

**Affiliations:** 1 Graduate Program in Immunology, University of Michigan, Ann Arbor, MI, United States of America; 2 Biostatistics Department, University of Michigan School of Public Health, Ann Arbor, MI, United States of America; 3 Pulmonary and Critical Care Medicine Division, Department of Internal Medicine, University of Michigan, Ann Arbor, MI, United States of America; 4 MedImmune, Gaithersburg, MD, United States of America; 5 Department of Internal Medicine, Weill Cornell Medical College, New York, NY, United States of America; 6 Department of Microbiology and Immunology, University of Michigan, Ann Arbor, MI, United States of America; University of Alabama at Birmingham, UNITED STATES

## Abstract

**Rationale:**

Biomarkers in easily accessible compartments like peripheral blood that can predict disease progression in idiopathic pulmonary fibrosis (IPF) would be clinically useful regarding clinical trial participation or treatment decisions for patients. In this study, we used unbiased proteomics to identify relevant disease progression biomarkers in IPF.

**Methods:**

Plasma from IPF patients was measured using an 1129 analyte slow off-rate modified aptamer (SOMAmer) array, and patient outcomes were followed over the next 80 weeks. Receiver operating characteristic (ROC) curves evaluated sensitivity and specificity for levels of each biomarker and estimated area under the curve (AUC) when prognostic biomarker thresholds were used to predict disease progression. Both logistic and Cox regression models advised biomarker selection for a composite disease progression index; index biomarkers were weighted via expected progression-free days lost during follow-up with a biomarker on the unfavorable side of the threshold.

**Results:**

A six-analyte index, scaled 0 to 11, composed of markers of immune function, proteolysis and angiogenesis [high levels of ficolin-2 (FCN2), cathepsin-S (Cath-S), legumain (LGMN) and soluble vascular endothelial growth factor receptor 2 (VEGFsR2), but low levels of inducible T cell costimulator (ICOS) or trypsin 3 (TRY3)] predicted better progression-free survival in IPF with a ROC AUC of 0.91. An index score ≥ 3 (group ≥ 2) was strongly associated with IPF progression after adjustment for age, gender, smoking status, immunomodulation, forced vital capacity % predicted and diffusing capacity for carbon monoxide % predicted (HR 16.8, 95% CI 2.2–126.7, P = 0.006).

**Conclusion:**

This index, derived from the largest proteomic analysis of IPF plasma samples to date, could be useful for clinical decision making in IPF, and the identified analytes suggest biological processes that may promote disease progression.

## Introduction

The classification of idiopathic interstitial pneumonia is given to a heterogenous group of interstitial lung diseases that are differentiated on the basis of their clinical, radiographic and pathologic features [1, 2]. Within this group of diseases, the most common and most deadly diagnosis is idiopathic pulmonary fibrosis (IPF)[3]. IPF can be diagnosed using a multidisciplinary approach looking at clinical and laboratory features corroborated either by characteristic high-resolution CT scan or by the presence of the usual interstitial pneumonia pattern of histopathology seen on lung biopsy [4, 5]. Even with definitive diagnosis, the natural course of IPF can vary significantly with some patients experiencing relative stability, while others experience more rapid disease progression [defined as a decline of 15% in diffusion capacity of the lung for carbon monoxide (DL_CO_) or 10% in forced vital capacity (FVC)] [6, 7] over 2–5 years. Well-validated biomarkers from peripheral blood could have tremendous impact if they could help to differentiate a diagnosis of IPF from other forms of interstitial lung disease (e.g hypersensitivity pneumonitis) that may respond to therapy or if they could provide accurate prognostic information about the IPF disease course to aid patients in deciding on medication options or clinical trial participation [6, 8, 9].

Many groups have reported on biomarker analysis in IPF using approaches such as genomic or transcriptomic profiling, microbiome analyses and candidate biomarker analyses in blood, bronchoalveolar lavage fluid and lung tissue specimens [9–14]. Blood-based biomarkers have been studied previously and have involved looking at leukocyte phenotypes, both by flow cytometry as well as gene profiling, and also at circulating mediators [9, 11, 14–19]. Interestingly, there is a growing appreciation that markers of altered immune function characterize IPF [14–16, 18, 19]. In several studies, markers of lymphocyte activation carry worse prognosis in IPF, while in other cases, deficiencies in immune activation are noted. These data, coupled with recent studies showing worse outcomes in IPF patients treated with the immunosuppressive regimen of prednisone, azathioprine and N-acetylcysteine [20] have led to the speculation that occult immune insults (e.g. infections) may drive at least some cases of IPF progression [21, 22]. This is also underscored by recent studies showing that progressive disease in IPF is associated with overall bacterial burden as well as certain classes of organisms [12, 13]. Such biomarker analyses have provided potential insight into the pathogenic mechanisms which may account for IPF disease progression.

Similarly, studies have explored the contribution of angiogenesis to fibrogenesis with mixed results [23]. Some human studies have shown increases in markers of angiogenesis in IPF [24–28], while others have shown elevations in angiogenesis inhibitors [29] or reduced levels of angiogenic factors [30, 31]. Some studies have reported mixed phenotypes measuring higher levels of both angiogenic and angiostatic factors in IPF [32, 33]. These variations highlight the fact that angiogenesis may be important for both pathologic fibrogenesis as well as tissue repair.

In terms of protease imbalances in IPF, several studies have identified alterations and these have been the subjects of several review articles. Alterations in metalloproteases are the most commonly noted [34, 35], but proteosomal processing has also been implicated in fibrotic lung disease [36].

Recently, a new technology has become available for use in biomarker discovery. The slow-off rate-modified aptamer (SOMAmer) SOMAscan panel [37]-based proteomics platform measures 1129 analytes with increased sensitivity for low-abundance biomarkers. Using this unbiased approach, we analyzed plasma from 60 IPF patients who were enrolled in the NIH-sponsored observational study, *COMET* (Correlating Outcome Measures to Estimate Time to progression in IPF) [16, 17] and who completed 80 weeks of follow up. We then correlated biomarkers with measures of disease progression and identified a six-SOMAmer analyte index composed of measures of the following proteins: soluble vascular endothelial growth factor receptor 2 (VEGFsR2), ficolin-2 (FCN-2), legumain (LGMN), cathepsin-S (cath-S), inducible T cell costimulator (ICOS) and trypsin-3 (TRY-3). Patients experienced disease progression when measures of VEGFsR2, FCN-2, LGMN and cathepsin S were below their identified thresholds (9559, 2015, 5173 and 1451 relative fluorescent units (RFU) respectively). Conversely, when levels of ICOS and TRY3 were measured above 8031 and 928 RFU respectively, the combination predicted IPF progression.

## Materials and Methods

### Patient population

Each site for the COMET study received local Institutional Review Board approval. The University of Michigan IRB approved the ancillary study as well. The COMET study was a multi-center, observational cohort study of well-defined IPF patients followed prospectively at 16 week intervals up to 80 weeks (clinicaltrials.gov, clinical trial ID no. NCT01071707). Patients were diagnosed as having IPF on the basis of characteristic computed tomography (CT) scans or usual interstitial pneumonia pathology confirmed by lung biopsy. All subjects underwent baseline assessment; including demographics, patient-reported descriptors; spirometry, DL_CO_, 6-minunte walk testing (6MWT) and high resolution CT. Patients were allowed to remain on current treatments. The primary outcome (combined endpoint) was progression-free survival as determined by the time until any of the following: death, acute exacerbation of IPF, lung transplant, or relative decrease in forced vital capacity (FVC, liters) of ≥10% or DL_CO_ (ml·min^−1^·mmHg^−1^) of 15%. Two previous studies have reported on data collected from the COMET cohort [13, 17].

Enrollment occurred from July 2010 through August 2012. Blood analyzed in this study was collected at week 0, near the time of diagnosis for the current study. Patient demographics and progressor status is shown in the Results section in Table 1 below. Because none of the patients in this study cohort died, all of our patients (both progressors and non-progressors were followed for about 80 weeks (518–645 days). This was an observational study and patients were allowed to remain on current treatments. The use of immunomodulatory medications was evaluated. These medications included prednisone, azathioprine and mycophenolate. The use of these medications in our study cohort was limited (8 patients in total, 13.3%). There were 2 patients (5.7%) on immunomodulation therapy in the progressor cohort and 6 patients (24%) on immunomodulation therapy in the non-progressor cohort. There was no significant difference between these 2 groups based on immunomodulation therapy (p = 0.057). Please see supplemental **Table A in the** S1 File.

**Table 1:** IPF Patient Characteristics for N = 60 COMET patients by 80-week Progression Status; Continuous Variables Reported as Mean (Standard Deviation), Categorical Variables Reported as N (%).

	All (N = 60)	Progressor (N = 35)	Non-progressor (N = 25)	p-value
**Age in Years**	64.6 (7.7)	65.2 (8.3)	63.7 (6.9)	0.48
**Male**	41 (68.3)	22 (62.9)	19(76.0)	0.28
**Smoker**				0.58*
**Never**	19 (31.7)	12 (34.3)	7 (28.00)	0.61**
**Past**	40 (66.7)	23 (65.7)	17 (68.00)	0.85**
**Current**	1 (1.7)	0	1 (4.00)	0.42**
**FVC, % pred**	70.0 (16.2)	71.2 (16.3)	68.2 (16.3)	0.5
**DLCO, %pred**	46.1 (13.1)	46.9 (12.9)	44.8 (13.8)	0.56

*Overall Chi-square comparison;

**Comparisons of corresponding category vs others.

### Sample preparation

Peripheral blood was collected in EDTA-containing vacutainers at study centers and samples were shipped by overnight mail using cold packs to the University of Michigan. Whole blood was centrifuged at 2500 rpm for 10 minutes and plasma was collected and frozen at -80^°^C in small aliquots. Samples were shipped to SomaLogics for analysis on the SOMAscan panel (1129 analytes). At the company, plasma was diluted at 3 different concentrations for analysis on the aptamer array at the optimal concentrations for each SOMAmer. An excel file containing the measurements for each analyte within the IPF patients at baseline is found in the Supplemental Materials S2 File.

### Statistical analysis

Each SOMAmer analyte is reported in relative fluorescent units (RFU) and is directly proportional to the amount of protein in the original plasma sample. Steps in constructing the disease progression index were as follows:

1. The ability of each of the 1129 continuous biomarkers to predict IPF progression status at 80 weeks was evaluated via ROC curves and a biomarker threshold was chosen to maximize the combined sensitivity plus specificity for each analyte. An estimated area under the curve (AUC) > 0.7 from a receiver-operating characteristic (ROC) analysis of the biomarker as a binary variable (above versus below its threshold) was required for further consideration in the following steps.
2. In order to further screen biomarker threshold variables, both unadjusted and adjusted odds ratios for 80-week progression were estimated separately via logistic regression. Adjustment factors included age, gender, smoking status, baseline percent predicted forced vital capacity (FVC) and baseline percent predicted diffusion capacity for carbon monoxide (DL_CO_). To be considered further for the IPF progression index, biomarker threshold variables had to maintain statistical significance at the 0.05 level in both unadjusted and adjusted analyses, and odds ratios from these models had to maintain the same direction of association.
3. To ensure independent prognostic ability of the biomarker threshold variables when used in combination, both multivariable logistic and Cox regression models were investigated. Based on the available sample size of 60 patients, a limit of four biomarker threshold variables in each multivariable model was enforced to prevent model instability. Automated model selection via the score method identified the top four binary biomarkers based on (a) multivariable logistic regression predicting 80-week progression status and (b) multivariable Cox proportional hazard regression predicting time to progression over the 80 week follow-up period. Between the two different models analyzed, 6 unique biomarker threshold variables were identified with p < 0.05 for predicting either 80-week progression status or time-to-progression. Thus, we chose to use the threshold measurements for these 6 analytes to create the IPF progression index.
4. Next, we wanted to give a relative weight to each biomarker for use in the index. Thus, for the 6-biomarker threshold variables used in the index, we estimated the difference in progression-free days during the first 80 weeks of follow-up using the area between Kaplan-Meier estimates of progression-free survival for patients with values above and below each biomarker threshold. These differences were used to generate a weighted numeric score.
5. For 4 of the biomarkers, progression was associated with values below the identified threshold, while for 2 markers, progression was associated with values above the threshold. However, we wanted to generate a score that would account for all 6 markers on a scale of positive values that were weighted according the Kaplan-Meier estimates for differences in progression-free days. To calculate this score, for each patient, if baseline levels of LGMN were below 5173.33 RFU, then the score got +3; if FCN2 levels were below 2015.33 RFU, then the score got +2; if VEGFsR2 levels were below 9559.30 RFU, then the score got +1; if Cath-S levels were below 1451.44 RFU, then the score got +1; if TRY3 measurements went above 928.22 RFU, then the score got +2; if ICOS levels went above 8032.61 RFU, then the score got +2. This generates a score for each patient accounting for all 6 biomarkers on a scale of 0 to 11.

Analyses evaluating the IPF progression index were then conducted. Three severity groups based on different scoring groups calculated using the index were created and evaluated via Cox regression and ROC analysis. Progression-free Kaplan-Meier survival curves for the 3 severity groups were displayed. Because we do not have access to a validation cohort of IPF patient plasma samples analyzed on the SOMAscan panel, we performed boot-strap analysis to determine how this index would theoretically perform in additional patient cohorts. Analyses were performed using SAS 9.4 (SAS Institute, Inc.), with plots created using R 3.2.0 (The R Foundation for Statistical Computing Platform).

There are 2 philosophies for accounting for multiple comparisons that are currently popular, the notion of restricting the false discovery rate or a Bonferroni-correction. The latter is considered overly conservative compared to the former. With approximately 50 statistical comparisons that occur after the AUC screening stage (step 1 above), maintaining a type I error of 5% via a Bonferroni adjustment would require tests to be statistically significant at the α = 0.001 level. Alternatively using a false discovery rate philosophy, on average 1 false discovery would be reported in this manuscript from the 50 comparisons if we require tests to be statistically significant at the α = 0.02 level.

## Results

### Patient demographics are similar in patients who progressed versus those that did not progress

Sixty IPF patients enrolled in COMET with longitudinal plasma samples collected at weeks 0, 48 and 80 were selected for analysis. However, results presented in this study only analyze plasma samples from the week 0 baseline time point to determine if biomarkers from this time point could predict disease progression over the next 80 weeks. Within this cohort, 35 (58%) met criteria for disease progression while 25 (42%) did not. There were no statistical differences in age, gender, and smoking history or baseline lung physiology between patients when categorized by progressor status (Table 1).

### Nine analytes predict IPF progression

Out of 1129 biomarkers analyzed, only 9 biomarkers satisfied criteria for estimated AUC > 0.7 from ROC analysis of the biomarker as a binary variable (above versus below its threshold); these were carbonic anhydrase 13, Cath-S, FCN2, granulin (GRN), ICOS, LGMN, nascent polypeptide-associated complex subunit alpha (NACA), TRY3 and VEGFsR2. Univariate ROC analyses for these 9 biomarkers are shown in Table 2, along with odds ratios (ORs) for progression for those above versus below their biomarker thresholds. Above threshold values of VEGFsR2, LGMN, FCN2, Cath-S and GRN were associated with lack of progression during follow-up (OR < 1.0, p < 0.05), and above threshold values for ICOS, TRY3, carbonic anhydrase 13 and NACA were associated with progression during follow-up (OR > 1.0, p< 0.05) in both adjusted and unadjusted analyses.

**Table 2:** Biomarker Threshold Values in RFU, Corresponding Sensitivity and Specificity for Predicting 80-week Progression Status and Univariate Odds Ratios (Unadjusted and Adjusted) for Progression When above Versus below the Threshold.

Biomarker	Better Prognosis Threshold (RFU)	Sensitivity	Specificity	AUC**	Unadjusted			Adjusted*		
					OR	95% CI	p-value	OR	95% CI	p-value
**VEGF sR2**	>9559.30	0.71	0.80	0.74	0.11	0.03–0.39	0.001	0.07	0.01–0.33	0.001
**LGMN**	>5173.33	0.54	0.96	0.74	0.04	0.005–0.32	0.003	0.04	0.00–0.38	0.005
**FCN2**	>2015.33	0.74	0.72	0.73	0.13	0.04–0.43	0.001	0.12	0.03–0.50	0.003
**Cathepsin S**	>1451.44	0.54	0.88	0.71	0.11	0.03–0.46	0.002	0.08	0.02–0.46	0.004
**ICOS**	<8031.61	0.77	0.64	0.71	6.00	1.93–18.68	0.002	13.50	2.88–62.88	0.001
**TRY3**	<928.22	0.77	0.64	0.71	6.00	1.93–18.68	0.002	6.10	1.65–22.82	0.007
**Carbonic anhydrase XIII**	<8738.54	0.69	0.76	0.72	6.91	2.16–22.10	0.001	5.90	1.64–20.98	0.007
**GRN**	>32046.22	0.69	0.72	0.70	0.18	0.06–0.55	0.003	0.11	0.03–0.50	0.004
**NACA**	<17976.83	0.69	0.72	0.70	5.61	1.82–17.33	0.003	7.50	1.57–35.88	0.012

Thresholds were chosen to maximize sensitivity plus specificity in separate ROC curve analyses. Odds ratios greater than 1.0 indicate higher risk of progression when above threshold; Odds ratios less than 1.0 indicate lower risk of progression when above threshold. Nine biomarkers met screening criteria of (1) AUC > 0.7, (2) unadjusted and adjusted P-values <0.05.

*Adjusted logistic models were adjusted by age, gender, smoking status, baseline FVC % predicted and DLCO % predicted.

**Abbreviations: AUC = Area under the ROC curve based on biomarker threshold variable binary threshold version of biomarkers; OR: Odds ratio; VEGFsR2 = soluble vascular endothelial growth factor receptor-2; LGMN = legumain; FCN2 = ficolin 2; ICOS = inducible T cell costimulator; TRY3 = trypsin 3; GRN = granulin; NACA = nascent polypeptide-associated complex subunit alpha.

### Determining analytes for scoring index

The top 4 biomarkers used to predict progression in 2 complementary multivariable model paradigms are shown in Tables 3 and 4, where biomarkers in Table 3 are selected using a multivariable logistic regression model for 80-week progression status and biomarkers in Table 4 are selected using a multivariable Cox proportional hazards model for time to progression. The logistic regression model identified the 4 best binary biomarkers as FCN2, VEGFsR2, Cath-S and TRY3. Cox proportional hazard regression model also placed FCN2 and TRY3 in its model along with ICOS and LGMN. Of the 6 unique biomarkers selected from the two multivariable progression model paradigms, values above identified thresholds were associated with lack of progression during the 80-week follow-up period in 4 cases (FCN2, VEGFsR2, Cath-S, LGMN) whereas 2 cases (ICOS and TRY3) were associated with progression.

**Table 3:** Best logistic regression model based on 4 binary biomarkers.

	Odds ratio	95% CI	P-value
**FCN2**	0.03	0.002–0.47	0.012
**VEGF sR2**	0.02	0.001–0.30	0.005
**Cathepsin S**	0.003	0.000–0.16	0.005
**TRY3**	50.10	2.93–857.24	0.007

**Table 4:** Best Cox proportional hazard regression model based on 4 binary biomarkers.

	Hazard ratio	95% CI	P-value
**LGMN**	0.27	0.13–0.56	0.0003
**FCN2**	0.49	0.22–1.08	0.076
**ICOS**	2.32	1.02–5.24	0.044
**TRY3**	2.32	1.03–5.20	0.042

### 6-analytes create a weighted scoring index to predict IPF progression

**Fig 1:**
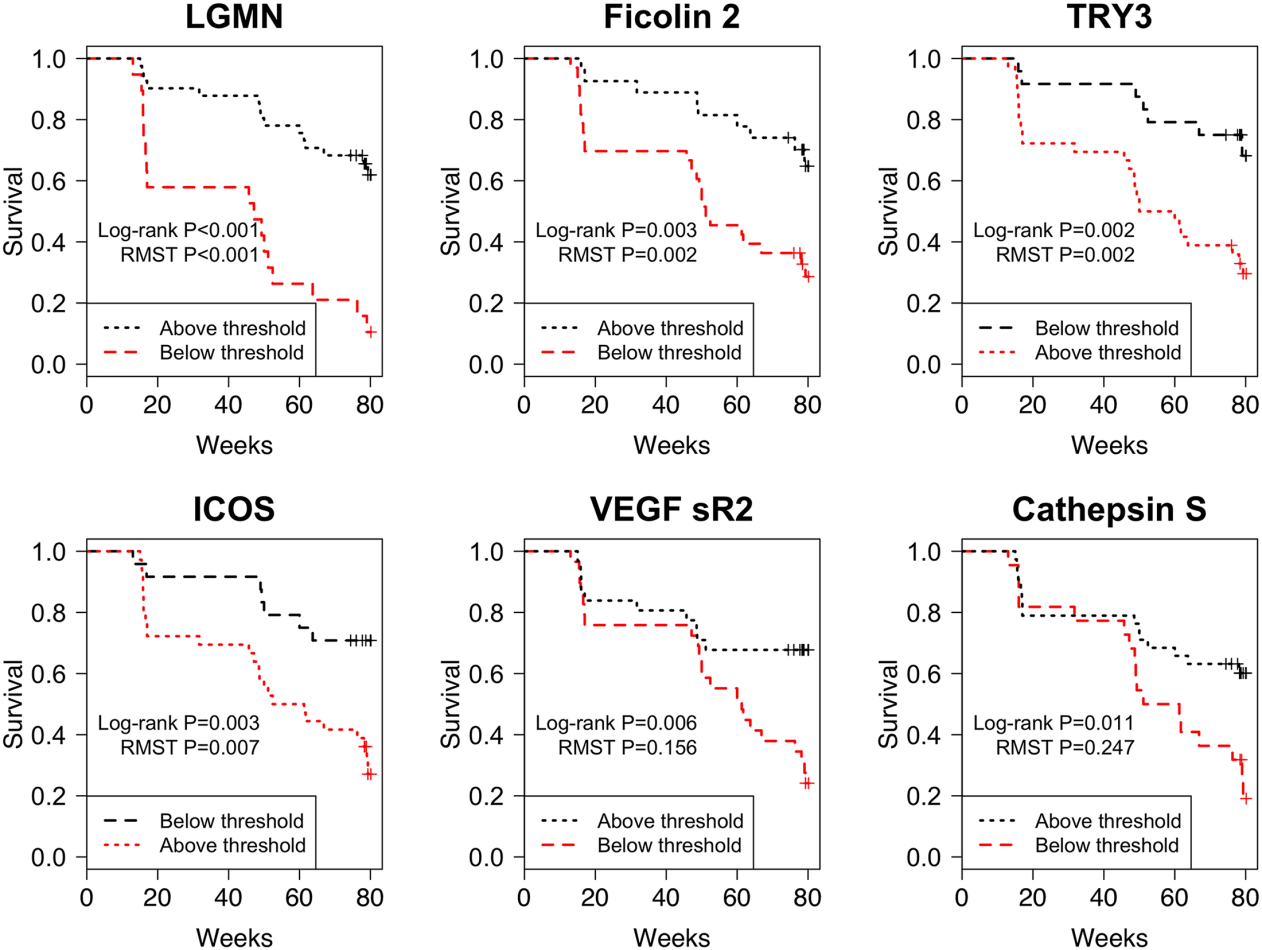
Kaplan-Meier curves showing progression free survival for IPF patients with baseline biomarker levels above or below the identified thresholds for 6 analytes best able to predict disease progression; these biomarkers are related to immune activation, protease function or angiogenesis.

All 6 binary biomarkers identified for the scoring index had different time-to-progression profiles, suggesting different weights should be used for each in predicting progression within the index. Kaplan-Meier curves in Fig 1 display progression-free survival for those above (black dashes) and below (red dashes) their corresponding threshold values. ICOS, LGMN, FCN2 and TRY3 progression-free profiles diverged quickly following baseline threshold measurements, while differences in progression-free survival diverged later, after 40-weeks of follow-up, for those above and below Cath-S or VEGFsR2 thresholds at baseline. **Fig A in the** S1 File shows progression-free survival curves for the 3 biomarkers (NACA, GRN and carbonic acid hydrolase XIII) not selected for use in the index.

ICOS stands for inducible T cell co-stimulator, VEGFsR2 stands for soluble vascular endothelial growth factor receptor 2, TRY3 is trypsin 3 and LGMN is legumain. RMST indicates the comparison of restricted mean survival time (i.e. area under the K-M curves) between the two groups.

We created a weighted index score based on estimated differences in 80-week progression-free survival for patients above and below each biomarker threshold (Table 5). Biomarkers yielding the smallest separation in progression-free survival days over the 80 weeks were assigned a score weight of 1 (53 days for Cath-S; 63 days for VEGFsR2). Differences in progression-free survival days for biomarkers ICOS, TRY3 and FCN2 were approximately 2-fold that of Cath-S and VEGFsR2, and were given a score weight of 2. The largest difference in progression-free survival days was between LGMN threshold groups and this value was approximately 3-fold that of Cath-S and VEGFsR2; accordingly, a score weight of 3 was assigned. For each patient, a composite score was created by summing Table 5 score weights for each of the 6 biomarkers that fell on the poor prognosis side of a threshold (final scale of 0 to 11).

**Table 5:** Progression-free survival (in days) for each individual biomarker over approximately 80 weeks of follow up. Differences in days gained for patients on the favorable side of the biomarker thresholds versus the unfavorable side of the thresholds were utilized to generate a standardized score.

Biomarker	Marker prognosis		Difference in Days	Score Index
	Good	Bad		
**LGMN**	474	295	179	3
**FCN2**	488	360	128	2
**TRY3**	493	367	126	2
**ICOS**	485	373	112	2
**VEGF sR2**	448	385	63	1
**Cathepsin S**	437	384	53	1

**Fig 2:**
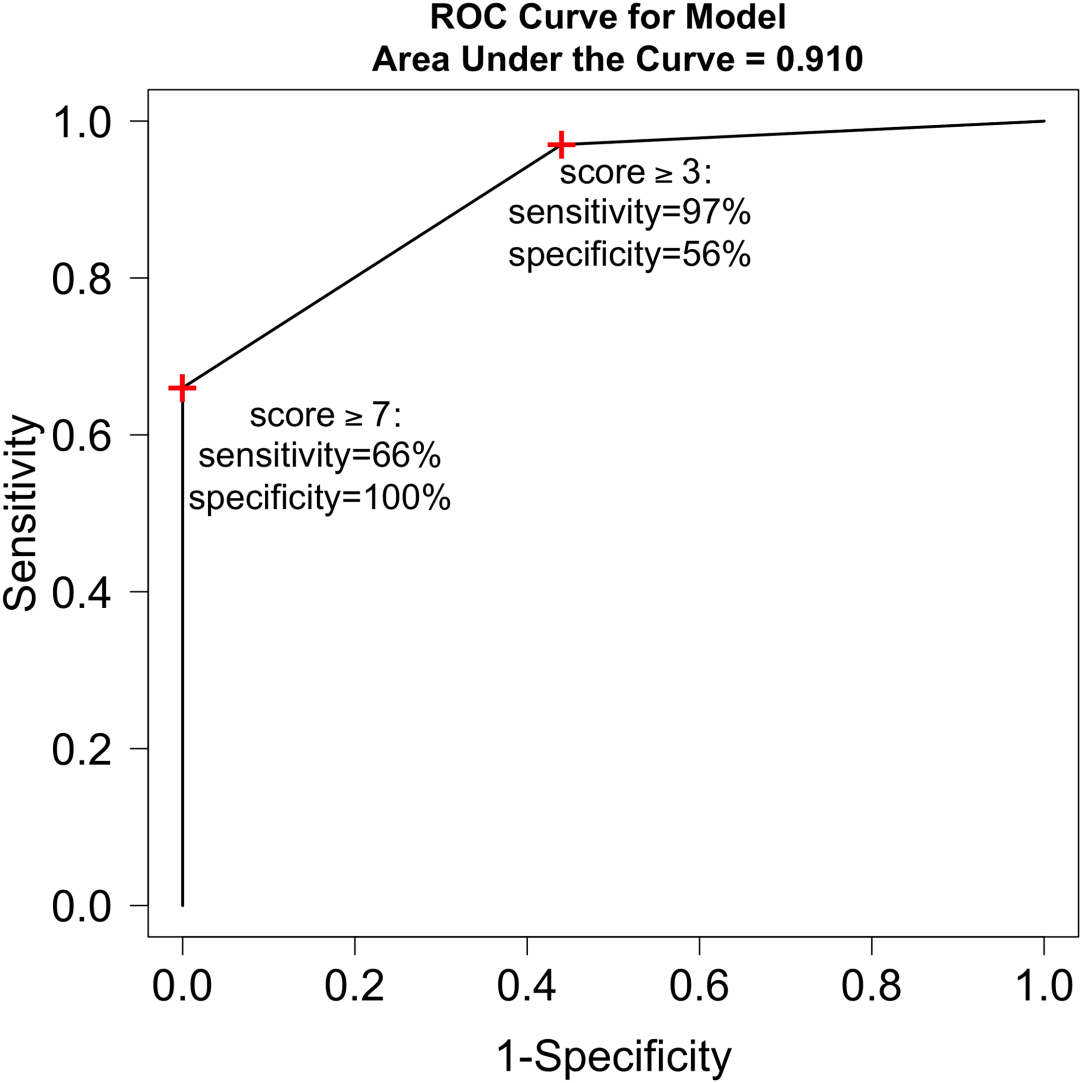
Receiver operating characteristic (ROC) curve using two index scoring thresholds (≥3 or ≥7) for prognostication. Higher areas under the curve indicate better overall classification, where AUC = 0.5 indicates a useless classification tool and AUC = 1.0 indicates a perfect classification tool. Our prognostic index score AUC = 0.91 indicates an extremely useful prognostic index score.

**Fig 3:**
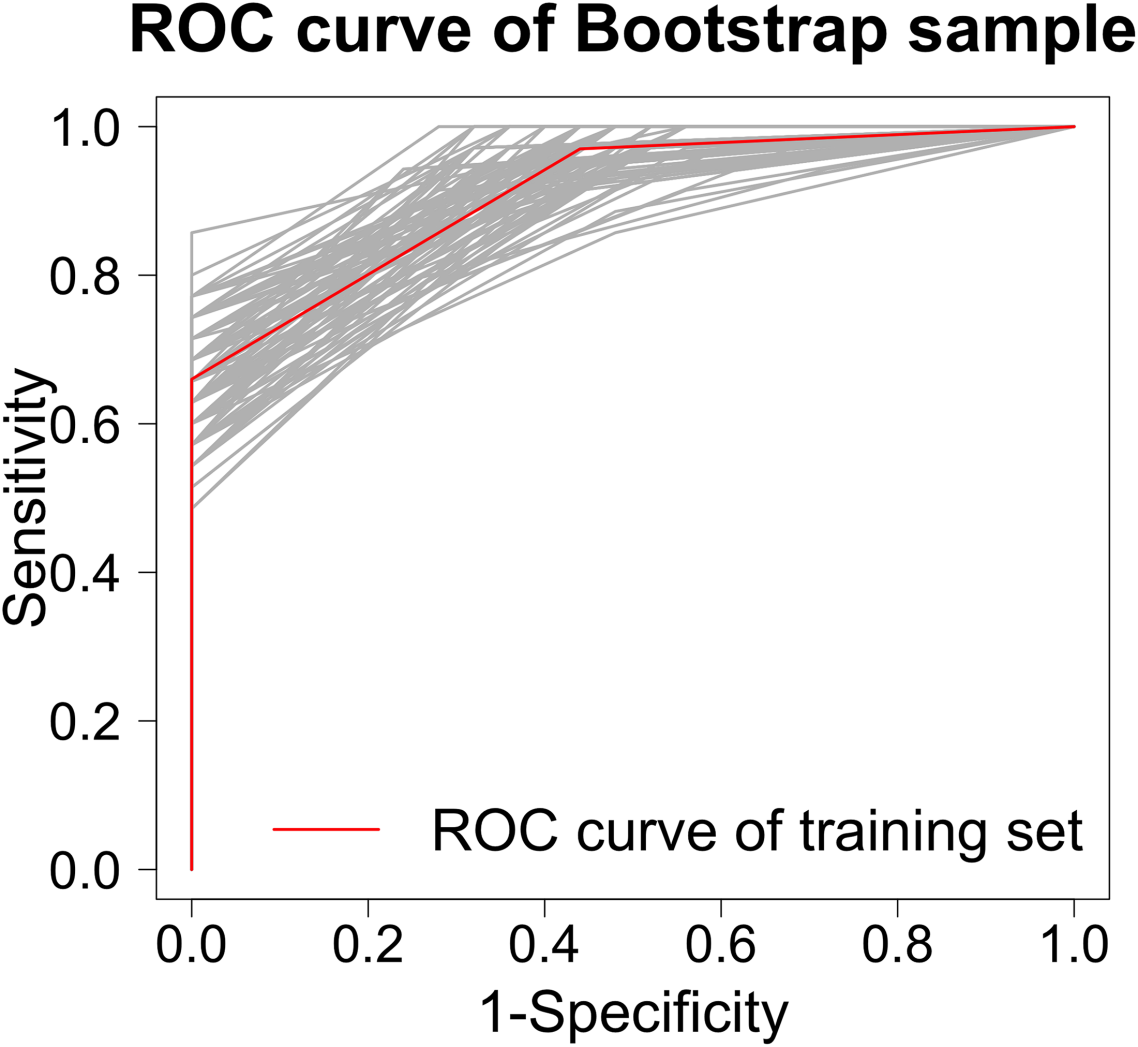
Bootstrapped distribution of estimated receiver operating characteristic (ROC) curve shown in Fig 2 (which is superimposed in red). 100 bootstrap samples were considered.

Table 6 shows the distribution of scores for progressors vs. non-progressors. Table 7 shows the distribution of scores categorized into 3 groups selected to represent different severities; group level 1 = scores 0–2, group level 2 = scores 3–6 and group level 3 = scores 7–11. The area under the ROC curve based on these 3 categorized levels was remarkably high (AUC = 0.91, Fig 2). Sampling variability of the estimated ROC curve is displayed in Fig 3 via bootstrap methodology; our cohort’s observed ROC curve is overlaid in this figure which analyzes 100 different bootstrap samples. A score of ≥3 on this index has 56% specificity and 97% sensitivity for predicting progression-free survival in IPF while a score of ≥7 had 66% sensitivity and 100% specificity for predicting progression-free survival in IPF.

**Table 6:** Distribution of scores for patients meeting the definition for progressor or non-progressor.

Frequency													
Row Pct													
Col Pct	Scores												
	0	1	2	3	4	5	6	7	8	9	10	11	Total
**Non-progressor**	5	2	7	4	4	1	2	0	0	0	0	0	25
	20	8	28	16	16	4	8						
	100	100	87.5	66.7	80	25	28.6						
**Progressor**	0	0	1	2	1	3	5	5	7	3	6	2	35
			2.9	5.7	2.9	8.6	14.3	14.3	20	8.6	17.1	5.7	
			12.5	33.3	20	75	71.4	100	100	100	100	100	
**Total**	5	2	8	6	5	4	7	5	7	3	6	2	60

**Table 7:** Distribution of group scores among progressors and non-progressors.

Frequency	Score groups			
Row Pct	1	2	3	Total
Col Pct	[0,2]	[3,6]	[7,11]	
**Non-progressor**	14	11	0	25
	56	44		
	93.3	50		
**Progressor**	1	11	23	35
	2.9	31.4	65.7	
	6.7	50	100	
**Total**	15	22	23	60

**Fig 4:**
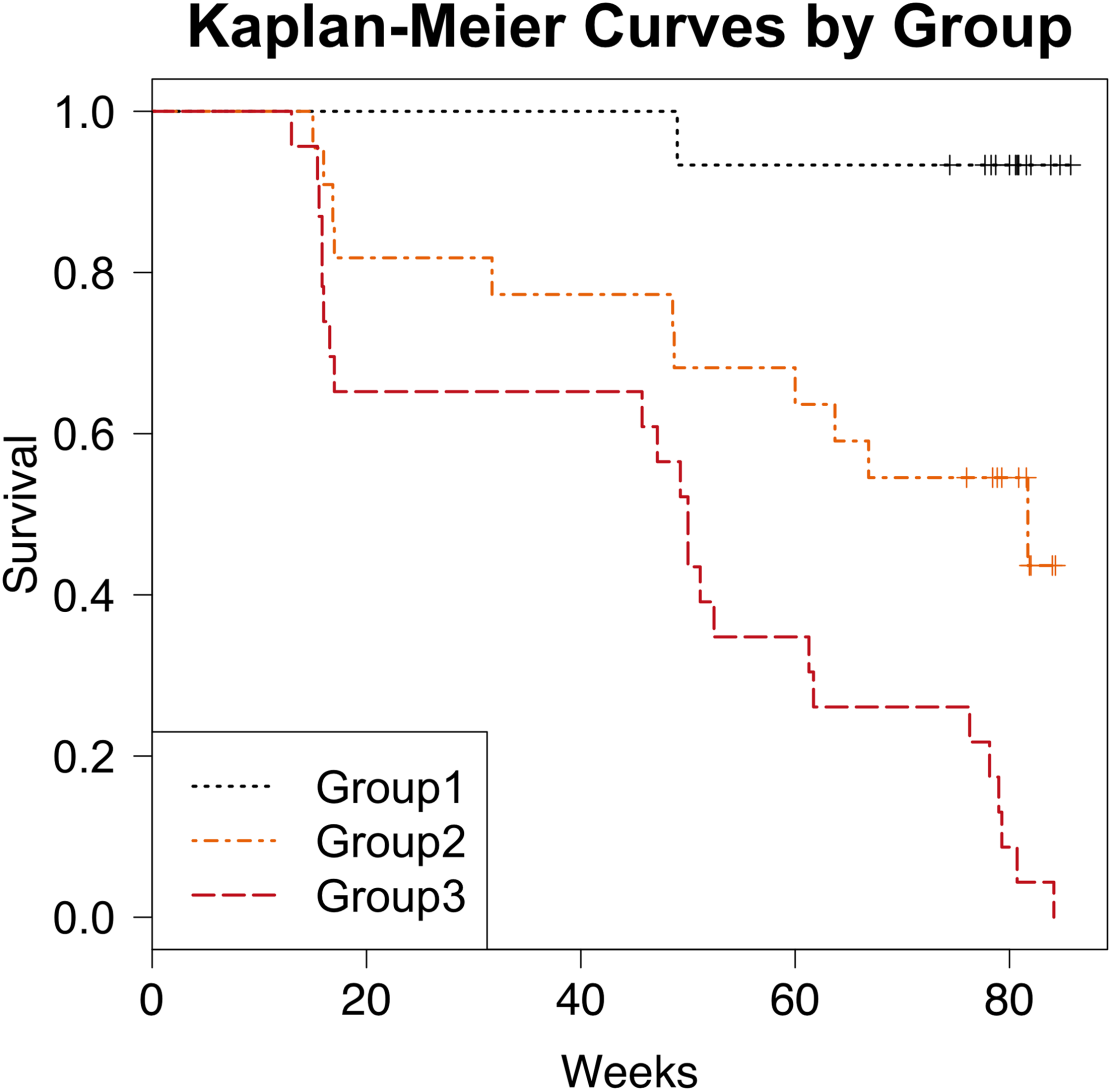
Kaplan-Meier curve showing progression free survival for patients according to the different severity groups in our weighted index score. In unadjusted analyses, A group level **increasing by 1** using this index has a hazard ratio = 4.02, (95%CI 2.28–7.10), P<0.0001 for predicting IPF progression by univariate Cox regression model. In adjusted analyses, a group level **increasing by 1** using this index has a hazard ratio = 4.06, (95%CI 2.17–7.60), P<0.0001 for predicting IPF progression by Cox regression model after being adjusted for age, gender, smoking status, baseline FVC percent predicted, baseline DLCO percent predicted and immunomodulation therapy.

**Fig 5:**
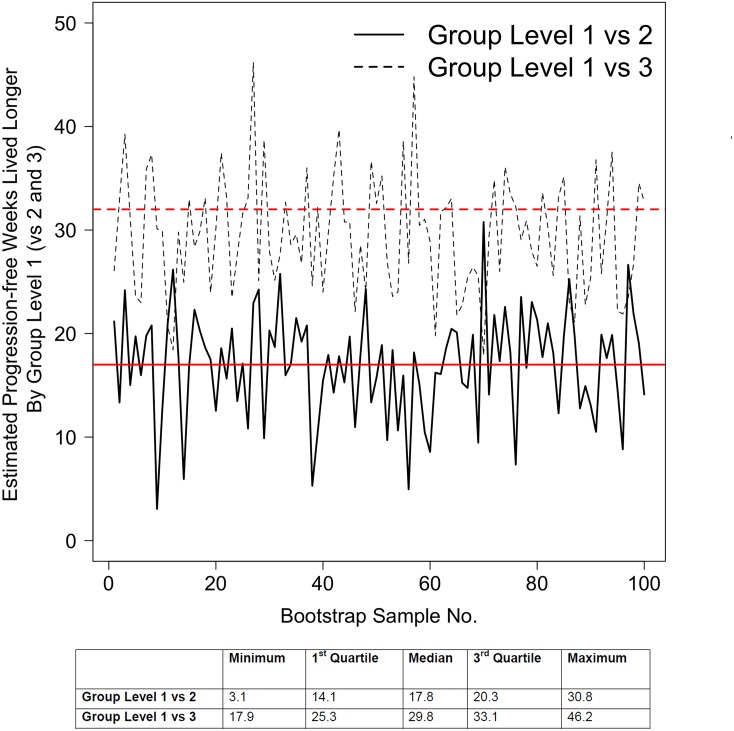
Sampling distribution of the number of progression-free weeks that group level 1 lived longer than group levels 2 and 3 over 80 follow-up weeks (Calculated via bootstrap methodology using 100 samples). Our cohort estimates are superimposed in red.

Fig 4 shows Kaplan-Meier survival estimates for the different groups (1,2 and 3) categorized in Table 7 to represent different severities. On average patients with scores in group 1 were progression-free approximately 17 weeks and 32 weeks longer than patients in groups 2 and 3, respectively, over approximately 80 weeks of follow-up (95% CI 6–28 weeks longer than group 2, p = 0.003; 95% CI 21–43 weeks longer than group 3, p<0.001). Sampling variability corresponding to the estimated number of weeks lived longer by patients in group 1 are displayed in Fig 5 via bootstrap methodology considering 100 samples. After adjusting for age, gender, smoking status, baseline FVC percent predicted, baseline DLCO percent predicted and immunomodulation, Group 2 [scores 3–6] and Group 3 [scores 7–11] have 9.1 and 29.0 times the hazard of Group 1 [scores 0–2] (95% CI for Groups 2 vs 1: 1.1, 71.4; p = 0.04; 95% CI for Groups 3 vs 1: 3.7, 250; p = 0.0013). A severity group level ≥ 2 [corresponding to an index score ≥ 3] has an adjusted hazard 16.8 times higher than group 1, (95% CI 2.2–126.7), P = 0.006. A group level increasing by 1 using this index has a hazard ratio = 4.06, (95%CI 2.17–7.60), P<0.0001 for predicting IPF progression by Cox regression model. Please note immunomodulation never shows significance in any of the models.

## Discussion

Studies have explored angiogenesis in fibrosis with mixed results. Some IPF studies showed elevations in angiogenesis inhibitors [29] or reduced angiogenic factors [30, 31]. Another study reports mixed phenotypes measuring higher levels of both angiogenic and angiostatic factors [33]. These variations suggest angiogenesis is important for both pathogenesis and repair. Regarding protease imbalances, alterations in metalloproteases are common [35], but proteosomal processing is also implicated in fibrotic lungs [36].

Blood-based biomarker analysis could be useful if it provides prognostic information for IPF patients. IPF natural history can vary considerably with some patients experiencing relative disease stability, while others experience rapid progression [6]. While there are two FDA-approved drugs available to treat IPF (pirfenidone and nintedanib), their costs are significant and improvement is limited to subsets of patients with mild to moderate functional impairment [38, 39]. Thus, the ability to accurately predict patients who are likely to progress could help focus treatment to patients at highest risk for functional decline.

Biomarkers may also determine aberrant signaling pathways associated with disease progression. Interestingly, some of our predictive analytes are associated with processes already known to be aberrant in IPF, namely immune dysfunction, angiogenesis and proteolysis. Accumulating evidence suggests IPF is characterized by immunologic alteration, with studies showing either activated leukocytes or impaired immunologic responses. Peripheral blood gene expression analysis demonstrated IPF patients are characterized by activated leukocyte phenotypes [15]. Similarly, CD4 T cells in IPF patients have activated phenotypes characterized by lower levels of CD28[18], but elevated levels of MHC class II, CD154 and oligoclonal Vβ gene expression [40]. Additionally, CXCR3+ CD8 cells which represent activated cytotoxic T cells also correlate with progression in IPF when found in increased percentages in peripheral blood [16]. There are conflicting studies showing both elevated levels of Tregs with an activated phenotype [19] but also reduced numbers of CD4+CD25+ cells in IPF [41, 42] with impaired Treg function [42]. We showed a correlation between increased percentages of CD14hi, CD16hi monocytes in circulation and IPF progression in COMET IPF patients [16] and these cells correspond to intermediate monocytes[43]. Thus, it is interesting that 3 of the SOMAmer analytes identified have known roles in immunologic functions namely, ICOS, FCN-2 and Cath-S.

ICOS is a CD28-superfamily costimulatory molecule upregulated on CD4+ T cells following antigenic stimulation. This molecule intensifies CD28 signaling during established immune responses and induces T cell effector functions [44]. In bleomycin-induced lung and skin fibrosis, ICOS-deficiency attenuated fibrosis [45]. Conversely, in IPF peripheral blood mononuclear cells (PBMCs), decreased expressions of ICOS, CD28 and lymphocyte-specific protein tyrosine kinase (LCK) are seen in more severe disease [15]. In our studies, elevated levels of ICOS measured in plasma correlated with worse IPF. While seemingly contradictory, these measurements in plasma were made in a different compartment than the earlier study looking at peripheral blood cellular mRNA levels. It is possible that the protein could be shed from activated cells into circulation, and that after activation of the cells, the mRNA levels would decrease. To determine if this is a feasible explanation, we asked whether ICOS is shed from T cells after activation; **Fig B in the** S1 File demonstrates activation of murine T cells releases ICOS into supernatant, suggesting that elevated ICOS in circulation could be an indicator of leukocyte activation. Certainly there is evidence for shedding of ICOS ligand induced by ICOS itself [46]. Thus, we hypothesize ICOS is released either by shedding or via exosomes upon cellular activation, and this may explain why lower levels of ICOS mRNA in PBMCs after activation [15], but higher levels of ICOS protein in plasma may both be associated with IPF progression.

Another factor believed to contribute to IPF pathogenesis is the presence of pathogens, both viral and bacterial. Studies in both IPF patients and animal models have shown viral infections, particularly herpesviral infections may promote fibrogenesis [reviewed in [22]]; however, bacterial burden may also predict worse outcomes [12, 13]. FCN-2 reductions correlate with IPF progression. Human L-Ficolin (FCN 2) is synthesized in liver and secreted into the bloodstream where it’s a major pattern recognition receptor [47]. FCN2 can opsonize several species of bacteria, in a manner similar to mannose-binding lectin (MBL) [48]. FCN2 and MBL activate the lectin complement pathway and studies have linked this pathway to fibrotic organ manifestations in Scleroderma, including ILD [49]. Furthermore, FCN2-deficiency may predispose patients to development of bronchiectasis [50]. Common variable immunodeficiency (CVID) patients with bronchiectasis also demonstrate low levels of FCN2 [51]. Additionally, mice lacking ficolin-A, the murine homolog of FCN2 showed increased mortality in a model of *S*. *pneumoniae* pneumonia [52, 53], suggesting a role for ficolin in *S*.*pnemoniae* infection. It is particularly noteworthy that microbiome analyses also conducted in COMET IPF patients suggest *Streptococcus* species are overabundant in progressive IPF patients [13]. Lower levels of FCN2 may contribute to overabundance of these bacterial species, which may, in-turn promote disease pathogenesis. *S*. *pneumoniae* promotes fibrogenesis through pneumolysin-mediated destruction of lung epithelial cells in animal models [54], suggesting a mechanism whereby bacterial burden may promote lung injury and fibrosis.

Cath-S is a single chain, non-glycosylated cysteine protease ubiquitously distributed in the lysosome. It is expressed mainly in lymphatic tissues and is characterized as a key enzyme in class II-mediated antigen presentation [55]. LGMN is a cysteine peptidase existing in a number of mammalian tissues, such as kidney, placenta, spleen, liver and testis. Interestingly, LGMN acts as a primary regulator of cysteine cathepsins [56]. Our study identifies an association between high circulating levels of both proteases and improved progression free survival in IPF. It is interesting to speculate that patients with higher levels of these proteases may be better able to activate immune cells to ward off such infections. This also implies that impaired endo-lysosomal function may directly promote fibrotic disease progression either through impaired immune activation or possibly other mechanisms as discussed below.

Inhibition of Cath S can promote autophagy and apoptosis [57]. Thus, lower levels of Cath -S in circulation could indicate IPF patients have aberrant cell survival pathways. Cath -S also promotes tumor cell invasion, metastasis and angiogenesis; once secreted into the extracellular milieu it causes degradation of extracellular matrix proteins including laminin, fibronectin, elastin, and some collagens [58]. Cath S ^-/-^ murine models demonstrate elevated expression of type 1 collagen and alpha-smooth muscle actin, with abnormal accumulation of autophagosomes in macrophages and impaired clearance of damaged mitochondria [59]. Interestingly, high levels of a natural cathepsin inhibitor, cystatin C, are found in IPF bronchoalveolar lavage fluid [60]. Thus, it’s not surprising that Cath -S levels are reduced in progressive IPF patients, and it is likely that this promotes fibrosis via multiple mechanisms.

LGMN was identified as diagnostic and prognostic liver fibrosis biomarker [61]. LGMN-deficient mice accumulate fibronectin and have worse outcomes in models of renal interstitial fibrosis [62]. Our study is the first to correlate lower levels of LGMN with poor outcomes in IPF, and we speculate LGMN may be important for degrading provisional matrix following injury.

Protease imbalances have been noted in IPF [34, 35]. Four isoforms of human trypsinogen protein are produced by alternative splicing. TRY3/mesotrypsin is a serine protease, encoded by the PRSS3 gene. Mesotrypsin/PRSS3 is overexpressed in human primary pancreatic cancer tissues and is associated with metastasis and poor prognosis of pancreatic [63], prostate, and non-small cell lung cancer [64, 65]. Alternative splicing produces four isoforms of human trypsinogen protein. How TRY3 influences IPF is unknown.

VEGF is a potent and specific endothelial cell mitogen that regulates blood and lymphatic vessel development and homeostasis. The VEGF receptor family consists of three members, VEGFR1 (FLT1), VEGFR2 (KDR/FLK1) and VEGFR3 (FLT4) [66]. Among these, VEGFR1 binds strongly to VEGF, VEGFR2 binds more weakly, and VEGFR3 shows essentially no binding, although it does bind to other members of the VEGF family. Soluble forms (VEGFsR1 and VEGsFR2) have been studied as potential biomarkers for a number of diseases but most of the data available are about VEGFsR1, not VEGFsR2 [67]. VEGFsR2 was first reported as a truncated 160KDa protein detected both in mouse and human plasma and an inverse correlation between the levels of VEGFsR2 and increasing tumor size was observed [68]. The SOMAmer reagent was selected against VEGFsR2 and our analysis indicates lower levels of this protein correlate with disease progression. It is unknown how circulating VEGFsR2 may correlate with endothelial cell activation or protective vs. pathologic angiogenesis in IPF, but we speculate lower levels of circulating VEGFsR2 would predict less ability to inhibit VEGF actions on endothelial cells, potentially enhancing angiogenesis associated with disease progression.

Our study is the first to analyze IPF patients using SOMAmers, and a limitation is small sample size and the lack of a validation cohort. The best approach to validate this screening panel would be an externally validated replication cohort in a larger group of IPF patients, and we hope publication of this work will encourage such a study. However, given the expense of SOMAscan^®^ validation on another cohort, we chose to address sensitivity and specificity of the index using bootstrap analysis. The index performs well across all 100 different bootstrap analyses performed (Fig 5) implying the index should perform well in other patient cohorts. Future studies are needed to determine whether smaller analyte panels could be cost-effective (something not currently available) or if ELISA measurements for these markers show similar or divergent results with the SOMAmer measurements. To get a sense of how well the SOMAmer measurements of a plasma protein would correspond with an ELISA-based measurement of the same protein, we measured periostin levels in the same patient samples using an ELISA developed by Abbott Pharmaceuticals. There is good correlation between measures made by SOMAmer and ELISA for this analyte with a Spearman correlation of r = 0.44, p<0.0001 suggesting that the SOMAmer assay is detecting native protein for this analyte (**Fig C in the** S1 File). This raises the possibility of whether we could use ELISAs for the 6 analytes to create a useful prognostic index. There are commercial ELISAs available for the 6 analytes we have identified, but it cannot be assumed that antibodies used in ELISAs to detect native protein would necessarily correspond to the same protein levels when measured by SOMAmer, which could potentially detect other forms of the protein. We anticipate that the SOMAmer technology will become more cost-effective with time, but our future studies will also focus on determining whether we can use an ELISA-based test for these 6 analytes to achieve similar prognostic accuracy.

There are generally two approaches to biomarker discovery. The first and most commonly used approach centers on a hypothesis. This approach is generally associated with strong biological rationale but is biased and time consuming. The other approach, as employed by our group in this study, is hypothesis free. This approach is rapid and involves the acquisition of large volumes of data with improved efficiency. However, one needs to be cognizant of the risk of false discovery. We chose to use the SOMAscan assay because of the large dynamic range of analytes at varying abundance (>8 logs of concentration) in a very small volume. Comparable proteomic assays would include mass spectrometry and ELISA platforms. Mass spectrometry is somewhat cumbersome and requires improved reproducibility and cost before it reaches mainstream clinical applicability. ELISA platforms are highly sensitive and useful for identifying single to multiple targets. However, it is now clear that these assays cannot be multiplexed above approximately 70 analytes without multiple measurements secondary to cross reactivity [69, 70].

This study is only capable of describing associations between the selected analytes and disease progression. As discussed above, we feel these analytes may be indicative of pathobiologic processes that could be aberrant in IPF, but it is also possible that these analytes are changed for reasons unrelated to disease. Unraveling this question will require future mechanistic work in animal models and using IPF tissues and cells.

Together, our results demonstrate a 6-analyte SOMAmer panel measuring circulating markers of immune function; proteolysis and angiogenesis can be used to create a simple index with excellent sensitivity and specificity for predicting progression-free survival in IPF. Use of this index on easily accessible plasma should be further validated to offer a simple way of predicting IPF patient outcomes. In addition, this analysis serves to highlight aberrant biological pathways associated with IPF disease progression, providing rationale for increased study of these biological pathways in lung fibrosis.

## Supporting Information

S1 File This file contains the following: **Table A) Immunodulation Therapy in the COMET Cohort. Fig A. Kaplan-Meier curves showing progression free survival for IPF patients with baseline biomarker levels above or below the identified thresholds (A)** Carbonic Anhydrase XIII, **(B)** Granulin(GRN**) and (C)** Nascent polypeptide-associated complex subunit alpha (NACA). RMST indicates the comparison of restricted mean survival time (i.e. area under the K-M curves) between the two groups. **Fig B. ICOS is shed by activated T cells.** A million CD4 positive splenocytes were stimulated with CD3 + CD28 then (TGFβ, 2ng/mL+ IL-6, 20ng/mL) or TH1 (IFNγ, 10ng/mL) for 24 hr. Cell free supernatants were collected and concentrated using Amicon Ultra Centrifugal filters (Millipore, Billerica, MA). Equal amounts of protein from each sample were separated on a 4–20% gradient SDS-polyacrylamide gel and transferred to a PVDF membrane (Amersham/GE Healthcare, Pittsburgh, PA). PVDF membrane was probed with rabbit monoclonal ICOS (Abcam). **Fig C. Periostin levels correlate on SOMAscan and ELISA.** The same plasma samples were run on a periostin ELISA developed by Abbot Pharmaceuticals and were compared to measures of periostin made by SOMAmer. The correlation was significant, Spearman r = 0.44; p<0.0001.
(DOCX)

S2 File This file contains the raw values for each SOMAmer analyte measured in IPF and control patients. (XLSX)
