# Supplementary material for: Six-SOMAmer Index Relating to Immune, Protease and Angiogenic Functions Predicts Progression in IPF
Source: PLoS One. 2016 Aug 4;11(8):e0159878. doi: 10.1371/journal.pone.0159878 (PMC4973878; doi:10.1371/journal.pone.0159878)
Supplement: S1 File — This file contains the following: Table A) Immunodulation Therapy in the COMET Cohort. Fig A. Kaplan-Meier curves showing progression free survival for IPF patients with baseline biomarker levels above or below the identified thresholds (A) Carbonic Anhydrase XIII, (B) Granulin(GRN) and (C) Nascent polypeptide-associated complex subunit alpha (NACA). RMST indicates the comparison of restricted mean survival time (i.e. area under the K-M curves) between the two groups. Fig B. ICOS is shed by activated T cells. A million CD4 positive splenocytes were stimulated with CD3 + CD28 then (TGFβ, 2ng/mL+ IL-6, 20ng/mL) or TH1 (IFNγ, 10ng/mL) for 24 hr. Cell free supernatants were collected and concentrated using Amicon Ultra Centrifugal filters (Millipore, Billerica, MA). Equal amounts of protein from each sample were separated on a 4–20% gradient SDS-polyacrylamide gel and transferred to a PVDF membrane (Amersham/GE Healthcare, Pittsburgh, PA). PVDF membrane was probed with rabbit monoclonal ICOS (Abcam). Fig C. Periostin levels correlate on SOMAscan and ELISA. The same plasma samples were run on a periostin ELISA developed by Abbot Pharmaceuticals and were compared to measures of periostin made by SOMAmer. The correlation was significant, Spearman r = 0.44; p<0.0001. (DOCX) [file pone.0159878.s001.docx]

**Supplemental Information**

**Table A. Immunodulation Therapy in the COMET Cohort.**

|  | **All** | **Progressor** | **Non-progressor** | **P-value** |
| --- | --- | --- | --- | --- |
| **Drug** | 8 (13.3) | 2 (5.7)  2 prednisone | 6 (24.0)  2 prednisone  3 azathioprine, prednisone  1 mycophenolate, prednisone | 0.06* |

*P value based on Fisher’s exact test.

**
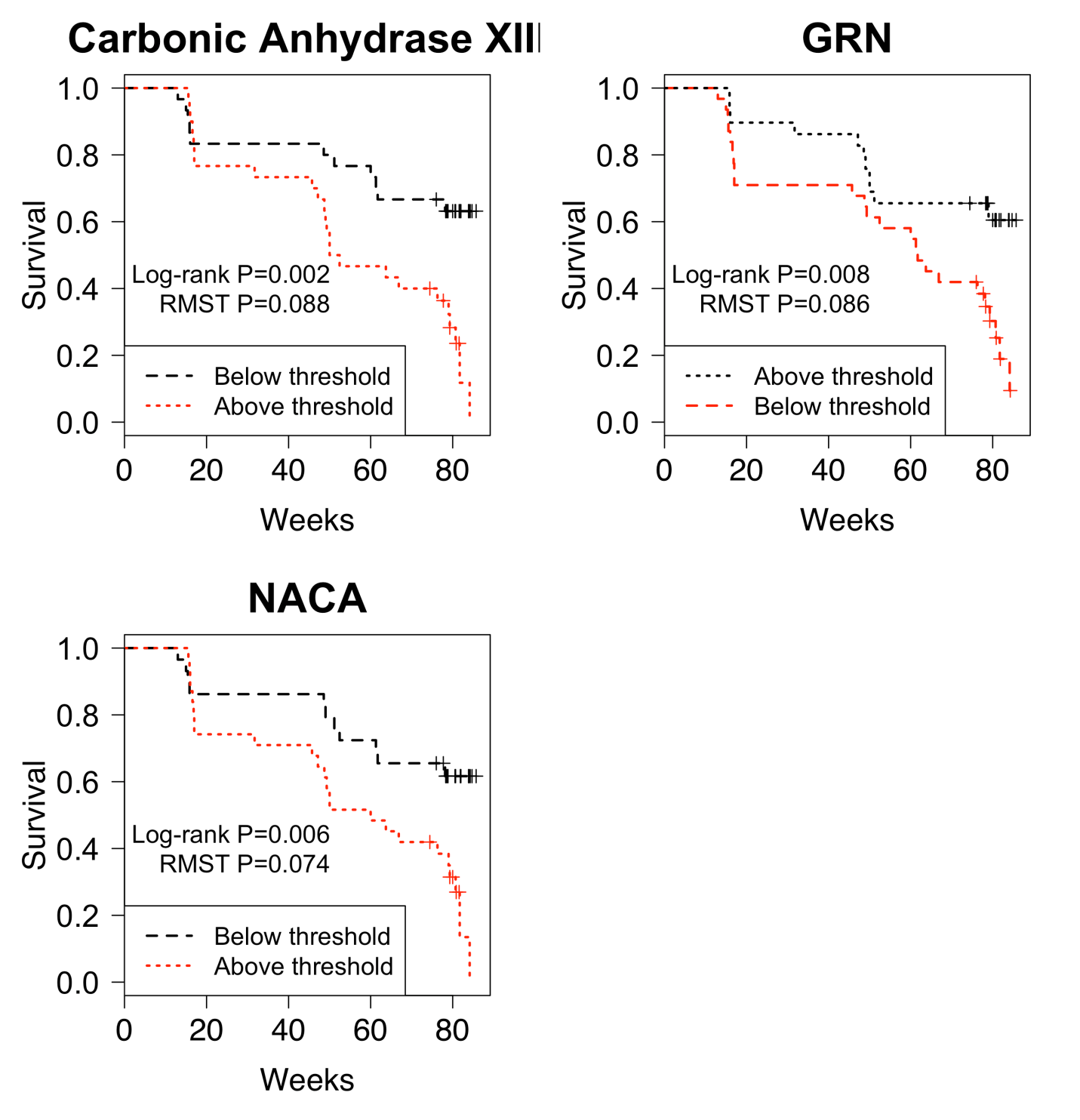
Fig A.** **Kaplan-Meier curves showing progression free survival for IPF patients with baseline biomarker levels above or below the identified thresholds (A)** Carbonic Anhydrase XIII, **(B)** Granulin(GRN**) and (C)** Nascent polypeptide-associated complex subunit alpha (NACA). RMST indicates the comparison of restricted mean survival time (i.e. area under the K-M curves) between the two groups.

**
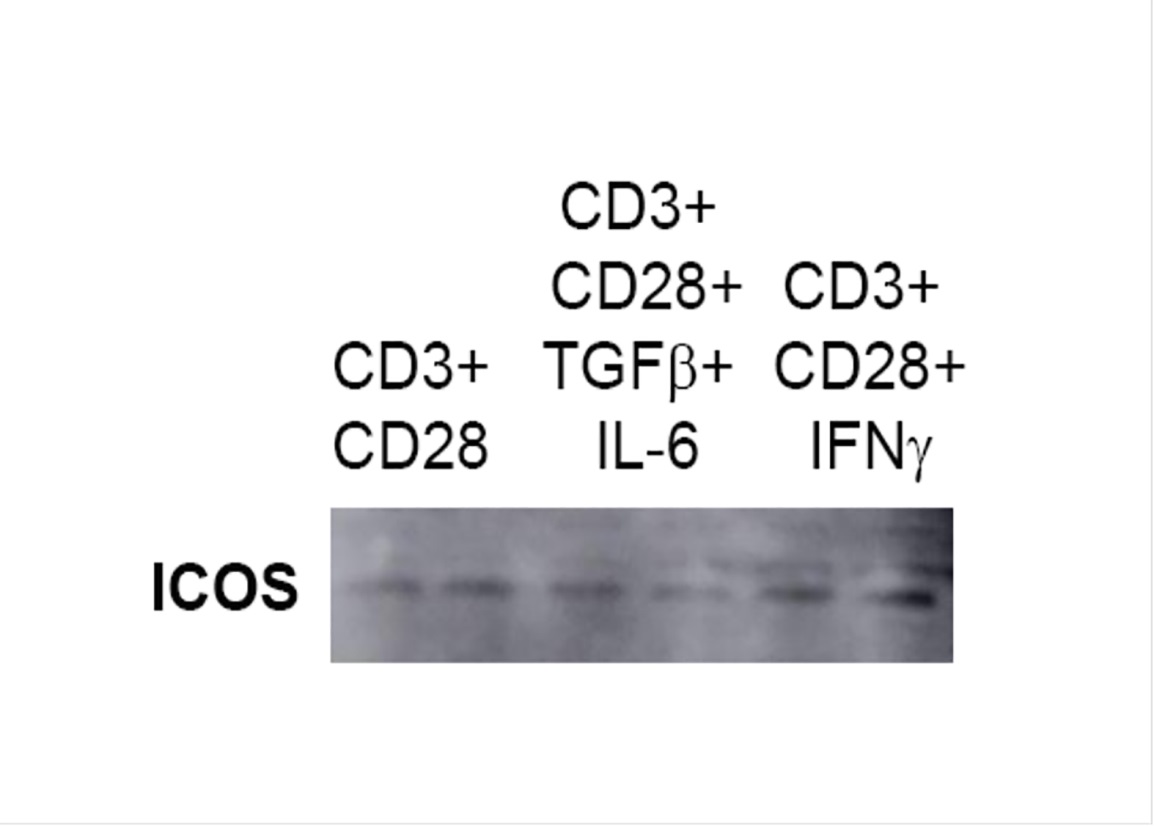
**

**Fig B. ICOS is shed by activated T cells:** A million CD4 positive splenocytes were stimulated with CD3 + CD28 then (TGFβ, 2ng/mL+ IL-6, 20ng/mL) or TH1 (IFNγ, 10ng/mL) for 24 hr. Cell free supernatants were collected and concentrated using Amicon Ultra Centrifugal filters (Millipore, Billerica, MA). Equal amounts of protein from each sample were separated on a 4-20% gradient SDS-polyacrylamide gel and transferred to a PVDF membrane (Amersham/GE Healthcare, Pittsburgh, PA). PVDF membrane was probed with rabbit monoclonal ICOS (Abcam).

**
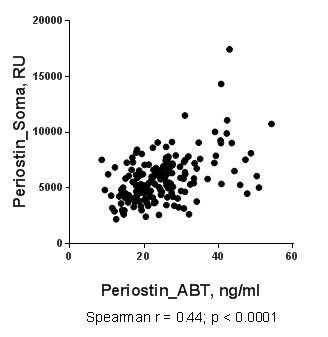
**

**Fig C. Periostin levels correlate on SOMAscan and ELISA:** The same plasma samples were run on a periostin ELISA developed by Abbot Pharmaceuticals and were compared to measures of periostin made by SOMAmer. The correlation was significant, Spearman r=0.44; p<0.0001.
